# Multi-Omics Profiling Suggesting Intratumoral Mast Cells as Predictive Index of Breast Cancer Lung Metastasis

**DOI:** 10.3389/fonc.2021.788778

**Published:** 2022-01-17

**Authors:** Leyi Zhang, Jun Pan, Zhen Wang, Chenghui Yang, Wuzhen Chen, Jingxin Jiang, Zhiyuan Zheng, Fang Jia, Yi Zhang, Jiahuan Jiang, Ke Su, Guohong Ren, Jian Huang

**Affiliations:** ^1^ Key Laboratory of Tumor Microenvironment and Immune Therapy of Zhejiang Province, Second Affiliated Hospital, Zhejiang University School of Medicine, Hangzhou, China; ^2^ Cancer Institute (Key Laboratory of Cancer Prevention &Intervention, National Ministry of Education), Second Affiliated Hospital, Zhejiang University School of Medicine, Hangzhou, China; ^3^ Department of Breast Surgery, Second Affiliated Hospital, Zhejiang University School of Medicine, Hangzhou, China; ^4^ Cancer Center, Zhejiang University, Hangzhou, China; ^5^ Department of Breast Surgery, The First Affiliated Hospital, Wenzhou Medical University, Wenzhou, China

**Keywords:** breast cancer, lung metastasis, tumor-infiltrating lymphocytes, mast cell, immunogenicity, risk prediction

## Abstract

Breast cancer lung metastasis has a high mortality rate and lacks effective treatments, for the factors that determine breast cancer lung metastasis are not yet well understood. In this study, data from 1067 primary tumors in four public datasets revealed the distinct microenvironments and immune composition among patients with or without lung metastasis. We used multi-omics data of the TCGA cohort to emphasize the following characteristics that may lead to lung metastasis: more aggressive tumor malignant behaviors, severer genomic instability, higher immunogenicity but showed generalized inhibition of effector functions of immune cells. Furthermore, we found that mast cell fraction can be used as an index for individual lung metastasis status prediction and verified in the 20 human breast cancer samples. The lower mast cell infiltrations correlated with tumors that were more malignant and prone to have lung metastasis. This study is the first comprehensive analysis of the molecular and cellular characteristics and mutation profiles of breast cancer lung metastasis, which may be applicable for prognostic prediction and aid in choosing appropriate medical examinations and therapeutic regimens.

## Introduction

Breast cancer is one of the leading causes of cancer death among women worldwide (1). Most breast cancer-induced deaths are caused by distant metastases which become lethal even after the primary tumors have been excised (2). However, at initial diagnosis, breast cancer patients often show rare signs of disseminated disease, yet up to 20% of patients ultimately develop distant metastasis (3). Breast cancer is prone to metastasize to the liver, bone, lung, brain and distant lymph nodes. Lung metastasis is reported to have a mortality rate of 60-70%, and current treatments for metastatic breast cancer are not that appropriate and effective (4). Advances in potentials drugs aiming at refraining lung metastasis have been seen (5), which surges the need to identify breast cancer patients who are more likely to develop lung metastases in order that they can benefit from early diagnosis following prevention treatments.

In clinical practice, the tumor node metastasis (TNM) staging system and the metastatic status of lymph nodes are standard diagnostic criteria for risk stratification in breast cancer patients. However, the clinical outcomes can be diverse for breast cancer patients with the same stage (6). It has been reported that nearly one-third of breast cancer patients that have not spread to the lymph nodes eventually develop distant metastases, and about one-third of breast cancer patients who develop lymph nodes metastases remain free of distant metastases 10 years after local treatment (7). The TNM staging system is primarily based on anatomy, which lacks biological changes that occurred in breast cancer. In addition, the mechanisms of hematogenous dissemination and lymphatic dissemination are dissimilar. These aforementioned reasons might explain the unsatisfactory prediction capability of the TNM staging system and the metastatic status of lymph nodes. Therefore, novel approaches to identify breast cancer patients who are prone to develop lung metastasis are needed.

Tumor metastasis consists of a cascade of complicated events, and successful metastatic colonization mainly relies on the inherent nature of the primary tumor cells (8). It has been reported that most metastatic drivers can be found in the primary tumor (8), and plenty of lung metastasis-related genes can facilitate both within the breast and the lung proliferation (9, 10). Primary tumors can also affect metastasis by regulating both systemic and secondary tumor microenvironment before and after dissemination (11). These findings indicate that we may prevent distant metastasis by targeting drivers of metastasis which are already existed in the primary tumors, which emphasizes the significance of analyzing data of primary tumors (12). In addition, it is of great clinical validity and potential utility for doctors to predict distant metastasis by evaluating primary tumors.

Tumor-infiltrating lymphocytes (TILs) can usually be found both in the stroma and the parenchyma of the tumor. TIL status has been proposed to independently predict patients’ prognosis, lymph nodes metastasis status, and treatment responses (13, 14). Generally, TILs are believed to reflect the mutation burden and the immunogenicity of tumors. However, the characteristics of immune infiltration in breast cancer metastasis to the lung and its relation with mutation burden are still unclear.

Mast cells are innate immune cells, which are characterized by their granules of inflammatory mediators, which are mainly known for their roles in allergic responses (15). Mast cells scatter in the stroma of breast tumors, and their functional and prognostic significances remain controversial, with evidence of both pro-and anti-tumoral roles (16).

Hence, the present study aimed to clarify the immune composition, hub genes, and mutational characteristics that drove breast cancer metastasis to the lung from public multi-omics datasets. Breast tumors that developed lung metastasis had distinct immune compositions, more aggressive malignant behaviors, severer genomic instability, higher immunogenicity but showed generalized inhibition of effector functions of immune cells. We also identified mast cell fraction as a prediction index of the status of lung metastasis in breast cancer patients. The low mast cell fractions defined breast tumors that were highly proliferative, with higher mutation burdens, and were prone to have lung metastasis.

## Materials and Methods

### Datasets Selection

To define the microenvironment characteristics of primary tumors that developed lung metastasis later, four published datasets with matched clinical and mRNA data were incorporated. The inclusion criteria being: (1) patients had intact mRNA and clinical data; (2) patients developed lung metastasis or without metastasis. 479 of 1302 breast cancer patients from the METABRIC in the European Genome-phenome Archive (https://www.ebi.ac.uk/ega/home, RRID: SCR_004944) were incorporated according to the inclusion criteria (17) (**Supplementary Table 1**). 448 of 1109 breast cancer patients from the TCGA dataset (https://portal.gdc.cancer.gov/, RRID: SCR_005012) were incorporated according to the same inclusion criteria (**Supplementary Table 1**). 82 and 58 patients from GSE2603 and GSE5327 in gene expression omnibus (GEO) (https://www.ncbi.nlm.nih.gov/geo/, RRID: SCR_005012) (9, 18) were also enrolled in this study (**Supplementary Table 1**). Demographics of the patients chosen for the study was shown in **Table 1**. The mutation data detected by the VarScan software (http://tvap.genome.wustl.edu/tools/varscan/, RRID: SCR_006849) of the TCGA cohort were downloaded from Genomic Data Commons Data Portal (GDC Data Portal) (https://portal.gdc.cancer.gov/, RRID: SCR_014514). We found matched mutation data of 399 breast cancer patients among 449 patients mentioned above.

**Table 1:** Demographics of the patients chosen for the study.

Variables	GSE2603 (n=82)	GSE5327 (n=58)	TCGA (n=448)	METABRIC (n=479)
Median age at diagnosis in years (IQR)	54.50 (46.75-64.00)	/	67.00 (60.00-71.00)	60.76 (51.09-69.37)
Median follow up time from diagnosis in days (IQR)	/	/	343.5 (114-1108)	3263 (1883-4672)
Lung metastasis status				
No metastasis	68	51	432	441
Lung metastasis	14	7	16	38
Pam50 subtype	/			
* Luminal A*		/	205	229
* Luminal B*			66	120
* HER2*			21	33
* Basal like*			100	68
* Normal breast-like*			14	29
* Unknown*			42	0
TNM stage	/			
* 1*		/	151	296
* 2*			281	172
* 3*			14	9
* 4*			2	2
ER status				
* Positive*	46	/	303	377
* Negative*	36		124	102
* Unknown*	0		21	0
PR status				
* Positive*	36	/	270	274
* Negative*	46		155	207
* Unknown*	0		23	0
HER2 status				
* Positive*	16	/	53	50
* Negative*	58		247	429
* Unknown*	8		148	0
Menopausal state	/			
* Pre*		/	84	103
* Post*			311	376
* Peri*			19	0
* Unknown*			34	0
Vital status				
* Alive*	/	/	434	284
* Dead*			14	195

PAM50, prediction analysis of microarray 50; ER, estrogen receptor; PR, progesterone receptor; HER2, human epithelial growth factor receptor 2; TNM, the tumor node metastasis.

The method of acquisition and application conformed to the guidelines and policies.

### Human Samples

Twenty tissue paraffin sections of breast cancer patients were obtained with approval from the ethics review committee of the Second Affiliated Hospital of Zhejiang University School of Medicine (IR2020001354) and each patient signed the informed consent (**Supplementary Table 2**). The study methodologies conformed to the standards set by the Declaration of Helsinki. The 8 samples among the no-metastasis group were with follow-up up to 10 years free of recurrences or distant metastasis.

### Calculation of Cell Abundance in the Tumor Microenvironment

The xCell algorithm was used to estimate the cell fractions of a tumor from its mRNA gene expression data (19). The relative abundance of 64 immune and stromal cell types of breast cancer patients in the above four cohorts was calculated.

### Gene Set Enrichment Analysis (GSEA)

To identify gene sets upregulated in the lung metastasis group, GSEA was performed with GSEA software (http://www.broadinstitute.org/gsea/, RRID: SCR_003199) from Broad Institute (20). Statistically significant cancer and metastasis-related gene sets (p < 0.05) were represented.

### Weighted Correlation Network Analysis (WGCNA)

The immune-related genes (IRGs) were retrieved from the immunology database and analysis portal (ImmPort) database (https://www.immport.org/home, RRID: SCR_012804) (21). WGCNA analysis was carried out with the transcription data of 1719 IRGs in the GSE2603 cohort. WGCNA was accomplished with the R package ‘*WGCNA’* (RRID: SCR_003302) (22, 23). Gene significance evaluated the relation of each gene with lung metastasis, and module membership indicated the correlation between gene expression profiles and module eigengenes. With a power = 4 as the optimal soft-thresholding power to ensure a scale-free co-expression network, a total of 7 non-grey modules were generated. Among these modules, the yellow module showed the highest correlation with breast cancer lung metastasis (**Supplementary Table 3**).

### Identification of Differentially Expressed Genes

82 breast cancer patients in the GSE2603 dataset were divided into two groups according to their metastasis status: the lung metastasis group and the no metastasis group. The genes with a p < 0.05 were defined as differentially expressed genes between the two groups. A total of 2,178 differentially expressed mRNAs (DEmRNAs) were found using the ‘*LIMMA’* package of R (24)(LIMMA, RRID: SCR_010943) (**Supplementary Table 4**).

### Mutation Landscape Visualization

The mutation data for 383 breast cancer patients without metastasis and 13 breast cancer patients with lung metastasis in the TCGA cohort were available. The synonymous variants were filtered out. The ‘*maftools’* package of R (25) was used for mutation spectrum visualization.

### Tumor Immunogenicity Analysis

The neoantigen load and its origin clonal information, mutation load, somatic copy number alterations (SCNAs) level, homologous recombination defects (HRD), intratumor heterogeneity, cancer testis antigens (CTA) score, T cell antigen receptor (TCR) evenness score, proliferation score, wound healing score, the number of segments, fraction altered, and aneuploidy score for each sample in the TCGA cohort were downloaded from the **Supplementary Data** of a previous study (26). The immunophenoscore (IPS) score for each sample in the TCGA cohort was obtained from The Cancer Immunome Database (TCIA) project (https://tcia.at/, RRID: SCR_014508) (27). For the neoantigen origin proteins that occurred only in the breast tumors metastasis to the lung, the association between these neoantigen origin proteins was investigated with the GeneMANIA plugin (http://genemania.org/, RRID: SCR_005709) in the Cytoscape software (28).

### Lung Metastasis Prediction

The receiver operating characteristic (ROC) curve was used to identify the optimal cut-off point and to stratify patients into the low mast cell group and the high mast cell group in four cohorts (**Supplementary Table 5**). In order to compare the survival difference between the two groups, the Kaplan-Meier (KM) survival analysis with a log-rank test was implemented in four cohorts. Meta-analysis (I^2^<30%, fixed-effects model) was performed to evaluate the predictive value of mast cell fraction in the pooled cohort. The ROC curves were implemented to evaluate the sensitivity and specificity of mast cell fraction, age at diagnosis, tumor size, positive lymph nodes number, grade, TNM staging system, and the signature reported by another study for predicting lung metastasis in four cohorts (29). The areas under the receiver operating characteristics curve (AUC) of the above predictive parameters were also calculated to compare the discriminatory capacity in four cohorts.

### Functional Enrichment Analysis

The Venn diagram was used to find the overlap between genes in the yellow module and DEmRNAs, which were regarded as hub genes for the subsequent functional enrichment analysis. Gene ontology (GO) enrichment analysis was performed using the ‘*clusterProfiler’* package of R (http://bioconductor.org/packages/release/bioc/html/clusterProfiler.html, RRID: SCR_016884) (30, 31).

### Immunohistochemistry (IHC)

Paraffin-embedded blocks were prepared, and 4-μm-thick sections were cut. Paraffin sections were deparaffinized, and antigenic retrieval was performed using Tris-EDTA (pH = 9) buffer in the thermostatic bath at 98° for 30 minutes (32). An IHC staining kit was used (Absin, Shanghai, China) as directed by the manufacturer. To evaluate the contents of mast cells in tumors, an anti-mast cell tryptase antibody was purchased from Cell Signaling Technology (E7M2U). All images were taken under the 10X objective lens by using Leica microsystems. Images of at least five random fields for each tumor sample were taken as TIFF files. Evaluation of immunohistochemical staining was carried out blinded to the clinicopathological characteristics.

### Repurposing of CMAP and FDA−Approved Drugs Against Breast Cancer Lung Metastasis

Drug repurposing of Broad Institute’s Connectivity Map (CMAP) (https://clue.io/, SCR_016204) drugs for the breast cancer lung metastasis was conducted on the genes common to the breast cancer lung metastasis-related gene list and the CMAP perturbation signatures (33). Breast cancer lung metastasis “signature” specific to the lung metastasis subgroup was chosen by selecting 150 significantly up-and down-regulated genes and hub genes mentioned above. The breast cancer lung metastasis signature was compared against drug perturbation signatures to find drugs that could reverse the breast cancer lung metastasis signature, that is to say, might be a potential therapeutic target for the breast cancer lung metastasis subgroup. The top 20 possible drugs were presented in the heatmap, which included the information about scores of 20 drugs’ effects on 9 cell lines, drugs names, and their descriptions. Negative scores (blue in the heatmap) indicated a competence for a given drug to reverse the breast cancer lung metastasis signature. The list of Food and Drug Administration (FDA)-approved drugs and their targets were downloaded from a former study (34).

### Statistical Analysis

All of the statistical analyses were conducted with the SPSS software (http://www-01.ibm.com/software/uk/analytics/spss/, RRID: SCR_002865) and R software (version 4.0.0; http://www.Rproject.org, RRID: SCR_001905). The continuous variables between two subtypes were compared using the two-sided Wilcoxon rank-sum test. The correlation between the two sets of quantitative data was calculated by the Spearman coefficient. All the tests were P<0.05, and two-sided was regarded as indicating significance unless otherwise stated.

## Results

### The Immune Landscape of the GSE2603 Cohort Suggested Breast Cancer Patients With or Without Lung Metastasis Had Distinct Immune Cell Infiltrations

**Figure 1:**
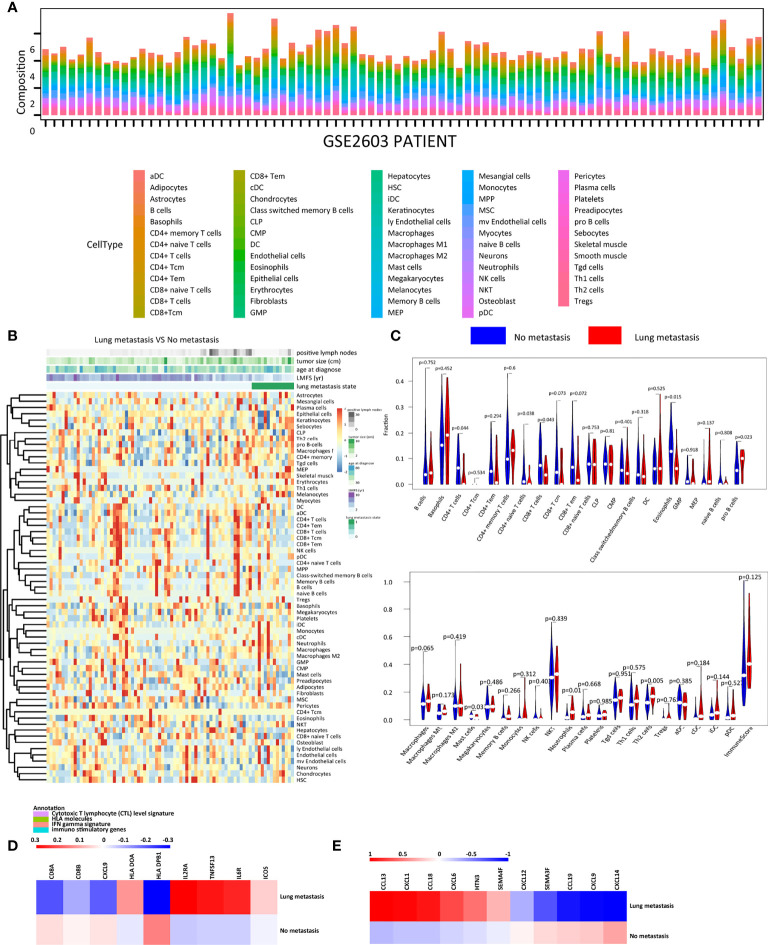
The immune landscape of the GSE2603 cohort. **(A)** Relative proportions of immune and stromal cell infiltrations in no-metastasis and lung-metastasis patients in the GSE2603 cohort. **(B)** Heatmap of the proportions of 64 cell types in no-metastasis and lung-metastasis patients in the GSE2603 cohort. **(C)** Violin plot showing the differences of each type of immune cell abundance between no-metastasis patients and lung-metastasis patients. Comparison of the mRNA expression fold changes of **(D)** cytotoxic T lymphocyte level signature, HLA molecules, IFN gamma signature, immuno-inhibitory genes, immuno-stimulatory genes, and **(E)** chemokines between no-metastasis and lung-metastasis patients. The fold change is the mRNA relative value to the mean of the whole cohort. Significantly differentially expressed genes were shown. DC, dendritic cells; MPP, multipotent progenitors; T_em_, effector memory T cells; CMP, common myeloid progenitors; MEP, megakaryocyte erythroid progenitors; GMP, granulocyte-macrophage progenitors; Tregs, regulatory T cells; HSC, hematopoietic stem cells; T_cm_, central memory T cells; mv endothelial cells, microvascular endothelial cells; ly endothelial cells, lymphatic endothelial cells; MSC, mesenchymal stem cells; aDC, activated dendritic cells; pDC, plasmacytoid dendritic cells; cDC, conventional dendritic cells; iDC, immature dendritic cells; Th2 cells, type 2 T helper cells; CLP, common lymphoid progenitors; Th1 cells, type 1 T helper cells; NKT, natural killer T cells; Tgd cells, gamma delta T-cells; CTL, cytotoxic T lymphocyte; IFN, interferon; HLA, human leukocyte antigen.

To explore the microenvironment of primary tumors with or without lung metastasis, we estimated the relative abundance of 64 immune and stromal cell types of breast cancer patients in the GSE2603 cohort (**Figures 1A, B** and **Table 1**) (19). The correlation analysis was performed to see the distribution pattern of 51 cell types in the tumor microenvironment (**Supplementary Figure 1**). Among TILs, CD4^+^ T cells, CD4^+^ naïve T cells, CD8^+^ T cells, eosinophils, and mast cells were found to be significantly enriched in breast cancer patients without lung metastasis, while pro B cells, neutrophils, and Th2 cells were significantly enriched in breast cancer patients with lung metastasis (**Figure 1C**). Immune genes related to cytotoxic T lymphocyte (CTL) signature, human leukocyte antigen (HLA) molecules, interferon (IFN) gamma signature, immuno-inhibitory genes, and immuno-stimulatory genes were specifically compared between the two groups (**Supplementary Figure 2**). Genes related to CTL and IFN gamma, including *CD8A*, *CD8B*, and *CXCL9*, expressed highly in the no metastasis group. Higher expression levels of HLA molecules like *HLA DOA* and *HLA DPB1*, together with immuno-stimulatory genes like *ICOS*, *IL2RA*, *IL6R*, and *TNFSF13* were found in the lung metastasis group (**Figure 1D**). The expression levels of chemokines in the two groups were also examined (**Supplementary Figure 2**). The lung metastasis group expressed higher levels of *CCL13*, *CCL18*, *CXCL1*, *CXCL6*, *HTN3*, and *SEMA4F*, while the no metastasis group expressed higher levels of *CCL19*, *CXCL12*, *CXCL14*, *CXCL9*, and *SEMA3F* (**Figure 1E**). The lung metastasis group and the no metastatic group have different chemokine profiles, which may be the cause of the varying immune cell infiltrations in these two groups.

### The Mutation Landscape of the TCGA Cohort Suggested Breast Cancer Patients With or Without Lung Metastasis Had Distinct Mutated Genes

**Figure 2:**
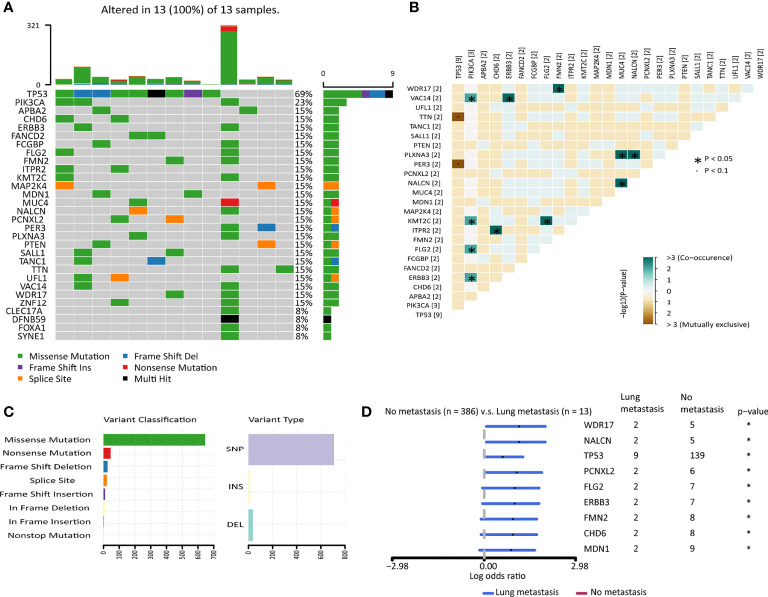
The mutation landscape of breast cancer patients with lung metastasis in the TCGA cohort. **(A)** Color-coded matrix of the top 30 most frequently mutated genes in breast cancer patients with lung metastasis (n=13). **(B)** Matrix of mutually exclusive or co-occuring mutational events. **(C)** Bar charts of variants classification and type. **(D)** Forest plot with x-axis as log10 converted odds ratio and differentially mutated genes between the no metastasis group and the lung metastasis group on the y-axis. *: p < 0.05.

Generally speaking, malignant tumors that were prone to have distant metastasis had higher mutation burdens (14). We plotted the heatmap of the top 30 highly mutated genes in the primary tumors of breast cancer patients developed lung metastasis in the TCGA cohort (**Figure 2A**). *TP53* was the most frequently mutated gene. *TP53* and *TTN*, *PER3* were detected to be mutually exclusive gene sets, while *PIK3CA*, *VAC14*, *KMT2C*, *FLG2*, *ERBB3* tended to co-occur. *MUC4*, *PLXNA3*, and *NALCN* also tended to co-occur. *ITPR2* and *CHD6*, *FMN2* and *WDR17* also formed co-occurring pairs (**Figure 2B**). Among the variants, missense mutation ranked first, followed by nonsense mutation and frameshift deletion. Single nucleotide polymorphisms (SNP) accounted for most of the variants (**Figure 2C**). Differentially mutated genes between the no metastasis group and the lung metastasis group were explored. 9 genes were significantly mutated highly in the lung metastasis group (**Figure 2D** and **Supplementary Table 6**), including *TP53*. However, there was no difference in the mutation load between the two groups (**Supplementary Figure 3A**). Though there was no difference in the mutation load between the breast cancer patients with or without lung metastasis, yet the two groups showed different mutation profiles.

### Breast Cancer Metastasis to the Lung Tends to Have More Malignant Characteristics and Less Intense Immune Responses

We then compared other parameters to find the factors involved in breast cancer metastasis to the lung. The SCNA level was higher in the lung metastasis group (**Supplementary Figure 3B**). HRD and intratumor heterogeneity were comparable in the two groups (**Supplementary Figures 3C, D**). Tumor mutation could generate neoantigens, thereby attracting immune cells to the tumor microenvironment. However, there was no difference in the single-nucleotide variant (SNV) neoantigen levels between the two groups (**Supplementary Figure 3E**). CTA, which was also able to stimulate immune responses, was found to be higher in the lung metastasis group (**Supplementary Figure 3F**). IPS scores, as an immune response predictor, were comparative in the two groups (**Supplementary Figure 3G**). The higher TCR evenness score in the lung metastasis group suggested the decreased diversity of TCR compared to the no metastasis group (**Supplementary Figure 3H**). The neoantigen origin proteins that occurred only in the lung-metastasis group were enriched in “cell-cell junction assembly”, “apical junction assembly”, and “cytoplasmic pattern recognition receptor signaling pathway” (**Supplementary Figures 4A, B**). Previously, adhesion and extracellular matrix molecules were reported to promote metastasis of disseminating cancer cells, including lung metastasis (35).

GSEA analysis of breast cancer patients in the GSE2603 cohort showed 13 significant terms associated with lung metastasis, including hallmarks of cancer “glycolysis”, “hypoxia”, “ATF4 activates genes in response to endoplasmic reticulum stress”, “abnormality of the mitochondrion”, “negative regulation of cell aging”, “positive regulation of cell migration involved in sprouting angiogenesis”, “positive regulation of blood vessel endothelial cell migration”, “unfolded protein response UPR”; lung metastasis-related pathways “mTORC1 signaling”, “SMID breast cancer relapse in lung up”; and pathways suggested failed immune responses “BTLA pos vs neg intratumoral CD8 T cell up”, “cellular response to dexamethasone stimulus”, “tumor escape from immune attack” (**Supplementary Figure 5**).

In general, breast cancer metastasis to the lung tended to have more malignant characteristics, yet failed to activate more intense immune responses.

### Immune-Related Hub Genes Participated in Breast Cancer Lung Metastasis

**Figure 3:**
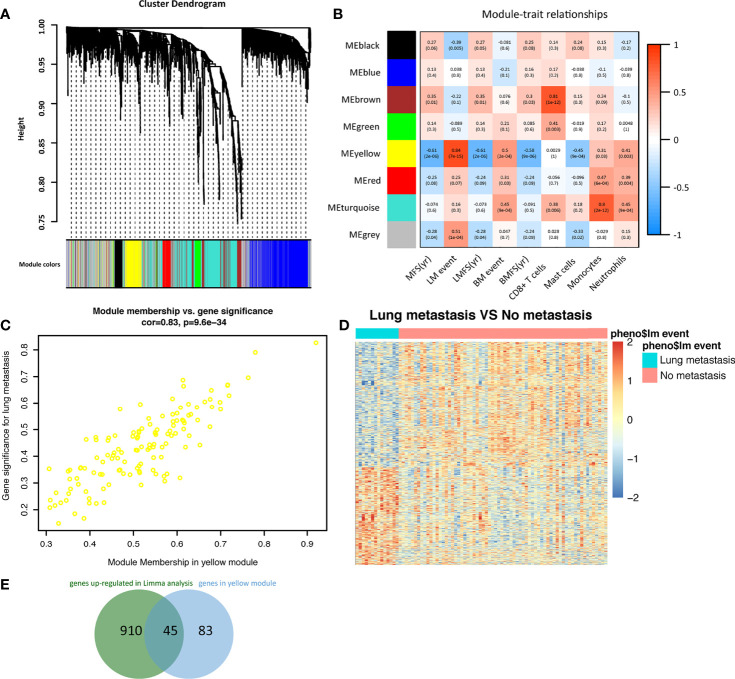
Identification of immune-related hub genes in breast cancer metastasis to the lung. **(A)** A dendrogram of the immune-related genes clustered based on different metrics. Each branch in the figure represented one gene, and every color below represented one co-expression module. **(B)** A heatmap presenting the correlations between the gene modules and clinical traits. The correlation coefficient in each grid represented the correlation between the gene module and the clinical trait, which decreased in color from red to blue. The yellow module showed the highest positive correlation with lung metastasis. **(C)** The gene significance for lung metastasis and module membership of the genes in the yellow module exhibited a high correlation of 0.83. **(D)** Heatmap of differentially expressed genes between breast cancer patients with or without lung metastasis in the GSE2603 cohort. **(E)** The Venn diagram indicated the overlap between differentially expressed genes and genes in the yellow module. LM, lung metastasis; LMFS, lung metastasis-free survival; MFS, metastasis-free survival; BM, bone metastasis; BMFS, bone metastasis-free survival.

WGCNA analysis was carried out with the transcription data of 1719 IRGs in the GSE2603 cohort. With a power = 4 as the optimal soft-thresholding power to ensure a scale-free co-expression network, a total of 7 non-grey modules were generated (**Figure 3A**). Among these modules, the yellow module showed the highest correlation with breast cancer lung metastasis (cor = 0.84, p = 7e^−15^) **(** **Figure 3B**). The scatter plot of module membership and gene significance depicted a significant correlation for each gene in the yellow module (cor: 0.83, p = 9.6e^−34^) (**Figure 3C**). Therefore, all 128 genes in the yellow module were considered to be highly correlated to breast cancer metastasis to the lung. We also performed DEmRNAs analysis between the breast cancer patients without or with lung metastasis in the GSE2603 cohort (**Figure 3D**). We found 2,178 differentially expressed genes, among which 955 were up-regulated in the lung metastasis group and 1,223 were down-regulated (**Supplementary Table 4**). Venn diagram was used to find the overlap between upregulated genes in the lung metastasis group and genes in the yellow module, which were regarded as hub genes for the subsequent functional enrichment analysis **(** **Figure 3E** and **Supplementary Table 7**). Tumor metastasis consists of a cascade of complicated events, including dissemination of cancer cells, arrest, adaptation to foreign tissue microenvironments, and metastases formation (36). During which, metastasizing cancer cells significantly adapt their metabolism (37). GO analysis of the hub genes was performed, which indicated that hub genes were involved in most of the above metastatic cascades, and thereby facilitating lung metastasis. (**Supplementary Figure 6A** and **Supplementary Table 8**). They facilitated vasculature development and hematogenous metastasis, promoted migration, created an immune-suppressive environment, rewired metabolism, and helped metastasizing cancer cells adapt to the lung microenvironment to form the metastases.

### Low Mast Cell Fraction Could Be an Indicator of Lung Metastasis in Breast Cancer Patients

**Figure 4:**
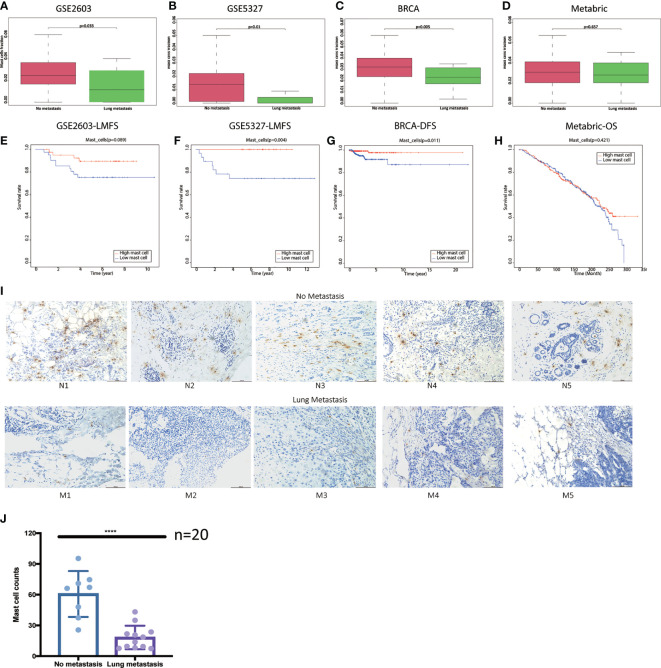
Breast cancer patients with lung metastasis have lower mast cell counts. Distributions of mast cell fractions with respect to lung metastasis status in **(A)** the GSE2603 cohort, **(B)** the GSE5327 cohort, **(C)** the TCGA cohort, and **(D)** the METABRIC cohort. **(E)** Representative images of IHC analysis of Tryptase protein in the 10 human breast invasive tumors. Scale bars, 100 μm. **(F)** Mast cell counts in the no metastasis group were significantly higher than the lung metastasis group. Student’s t test was used to analyze the significant differences. Kaplan-Meier curves of LMFS of breast cancer patients stratified by mast cell fraction in the **(G)** the GSE2603 cohort and **(H)** the GSE5327 cohort. Kaplan-Meier curves of DFS of breast cancer patients stratified by mast cell fraction in the **(I)** TCGA cohort. Kaplan-Meier curves of OS of breast cancer patients stratified by mast cell fraction in the **(J)** METABRIC cohort. BRCA, breast invasive carcinoma; METABRIC, molecular taxonomy of breast cancer international consortium; TCGA, the cancer genome atlas; N1-5, patients without metastasis 1-5; M1-5, patients with lung metastasis 1-5; DFS, disease-free survival; OS, overall survival; LMFS, lung metastasis-free survival; ****: p < 0.0001.

Compared to the no metastasis group, we found mast cell fractions were significantly lower in breast cancer patients with lung metastasis in the GSE2603 cohort (**Figure 4A**), the GSE5327 cohort (**Figure 4B**), and the TCGA cohort (**Figure 4C**). The mast cell fractions in the two subgroups of patients in the METABRIC cohort were the same (**Figure 4D**). And the patients with lower mast cell contents tended to have poorer prognoses in the GSE2603 cohort (**Figure 4E**), the GSE5327 cohort (**Figure 4F**), the TCGA cohort (**Figure 4G**), yet was unable to be statistically significant in the METABRIC cohort (**Figure 4H**). In addition, the mast cell fraction was significantly positively correlated to the lung metastasis-free survival in the GSE2603 cohort and showed a similar trend in the GSE5327 cohort (**Supplementary Figures 6B, C**). Similar analyses were done on other types of immune cells, but mast cells showed the greatest consistency in four cohorts (data not shown).

**Figure 5:**
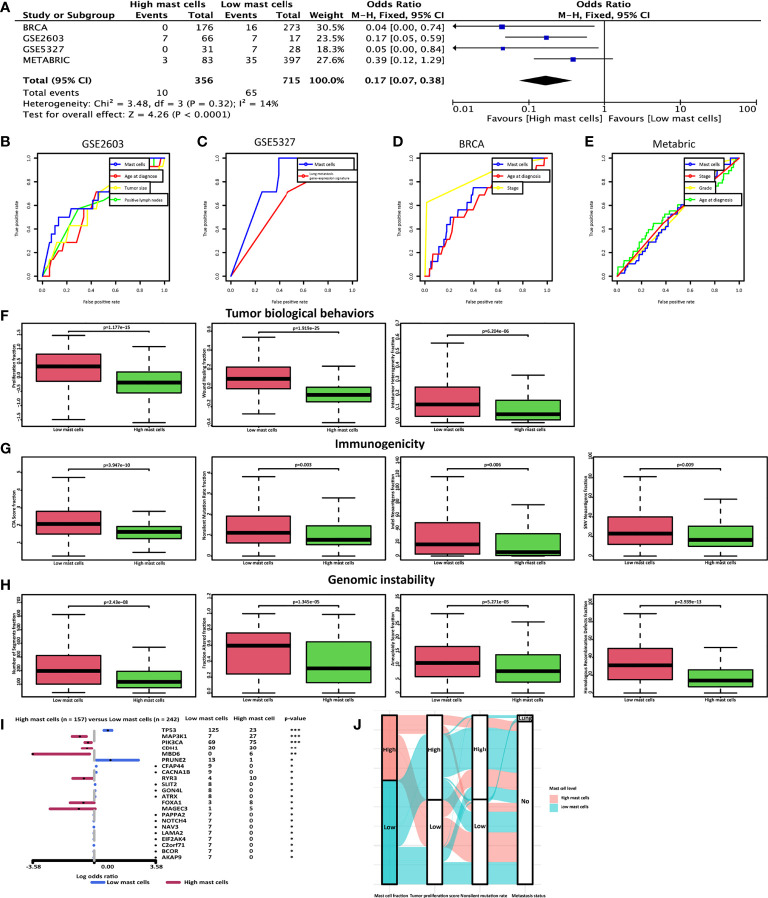
Low mast cell fraction could be an indicator of lung metastasis in breast cancer patients. **(A)** Meta-analysis was performed to calculate the pooled OR of mast cell fraction. ROC curves of mast cell fraction, age at diagnosis, tumor size, positive lymph nodes number, grade, TNM staging system, and signature reported by another study (29) in predicting lung metastasis in **(B)** the GSE2603 cohort, **(C)** the GSE5327 cohort, **(D)** the TCGA cohort, and **(E)** the METABRIC cohort. The AUC of the mast cell fraction for lung metastasis risk prediction was 0.682 in the GSE2603 cohort, 0.798 in the GSE5327 cohort, 0.708 in the TCGA cohort, and 0.521 in the METABRIC cohort. Distributions of **(F)** proliferation score, wound healing score, and intratumor heterogeneity **(G)** CTA score, mutation burden, and neoantigens **(H)** the number of segments, fraction altered, aneuploidy score, and HRD score with respect to mast cell-based subtypes. **(I)** Forest plot with x-axis as log10 converted odds ratio and differentially mutated genes between the high- and the low-mast cell groups on the y-axis. **(J)** A Sankey plot was used to reveal the correlations between mast cell fraction, tumor proliferation score, non-silent mutation rate, and lung metastasis status. BRCA, breast invasive carcinoma; METABRIC, molecular taxonomy of breast cancer international consortium; TCGA, the cancer genome atlas; OR, odds ratio; ROC, receiver operating characteristic; CTA, cancer testis antigens; HRD, homologous recombination defects; Indel, insertions and deletions; SNV, single-nucleotide variant; AUC, area under the receiver operating characteristics curve; *: p < 0.05; **: p < 0.01; ***: p < 0.001.

20 tumor sections of breast cancer patients without metastasis after 10-year follow-up and breast cancer patients with lung metastasis were used to evaluate the mast cell contents. Tryptase is one of the most abundant secretory granule-derived serine contained in mast cells and is a sensitive and specific marker for the localization of mast cells in tissues (16, 38). Mast cells are located both in the peritumoral adipose tissue and closed to the tumor cells (**Figure 4I**). As we reported before, mast cell levels in the no metastasis group were significantly higher than in the lung metastasis group (**Figure 4J**). We hypothesized that low mast cell fraction may act as an indicator of lung metastasis in breast cancer patients. Meta-analysis of the pooled cohort was performed to evaluate whether the low mast cell level was a promising marker for lung metastasis prediction. The result demonstrated that among the 1067 patients, those with lower mast cell fractions exhibited higher risks of lung metastasis than those with higher mast cell fractions (pooled OR = 0.17, 95% CI 0.07–0.38) (**Figure 5A**). The AUC of the mast cell fraction for lung metastasis risk prediction was 0.682 in the GSE2603 cohort (**Figure 5B**), 0.798 in the GSE5327 cohort (**Figure 5C**), 0.708 in the TCGA cohort (**Figure 5D**), and 0.521 in the METABRIC cohort (**Figure 5E**). It was comparable or even superior to other parameters used in metastasis prediction like tumor size, positive lymph nodes number, grade, TNM staging system, and signature reported by another study (18). Then we used mast cell fractions to stratify breast cancer patients into two subtypes (subtype 1: low mast cell group and subtype 2: high mast cell group). We found the proliferation score, the wound healing score, and intratumor heterogeneity were all significantly higher in the low mast cell subtype (**Figure 5F**). The low mast cell subtype also had higher CTA, higher mutation burden, and higher neoantigen loads, which indicated higher immunogenicity in this subtype (**Figure 5G**). Genomic instability-related parameters were also higher in subtype 1, including the number of segments, fraction altered, aneuploidy score, and HRD score (**Figure 5H**). Differentially mutated genes between the two subtypes were explored. *TP53* was significantly more frequently mutated in the low mast cell subtype (**Figure 5I**). In short, the low mast cell fraction defined a subtype of breast tumors that were highly proliferative, with higher mutation burdens, and were prone to develop lung metastasis (**Figure 5J**).

### Pro-Metastasis IRGs Were Significantly Negatively Correlated With Mast Cell Fractions

The estrogen-driven CXCL12 was reported to recruit mast cells (39). CXCL12 level was significantly low in the lung metastasis group (**Figure 1E**), and there existed a positive correlation between CXCL12 expression level and mast cell fraction through correlation analysis (**Supplementary Figure 7**). In addition, the expression levels of pro-metastasis IRGs we identified above were significantly negatively correlated with mast cell fractions, including MYC, AKT1, MAP2K1, NCK1, PLAUR, PSAT1, and S100A9 (**Supplementary Figure 7**).

### Drug Repurposing for Targeting Breast Cancer Metastasis to the Lung

**Figure 6:**
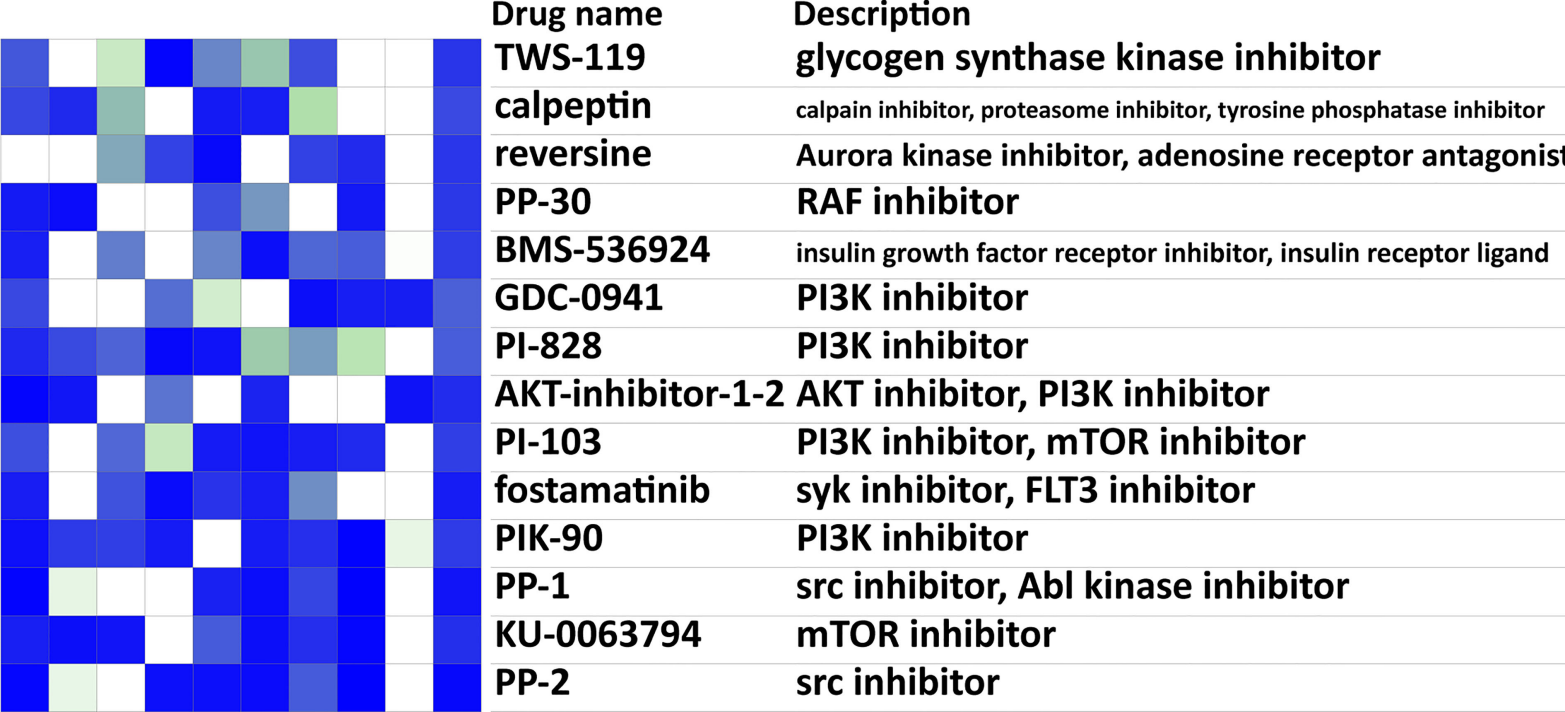
Drug prediction analysis for targeting lung metastasis. Heatmap of the top 20 possible CMAP drugs that could reverse the breast cancer lung metastasis signature. The scores of 20 drugs’ effects on 9 cell lines, drugs names, and their descriptions were shown. Negative scores (blue in the heatmap) indicated an ability for a given drug to reverse the breast cancer lung metastasis signature. CMAP, connectivity map.

We reported earlier that breast cancer metastasis to the lung tended to have more aggressive malignant characteristics but showed generalized inhibition of effector functions of immune cells. The current immune checkpoint blockade (ICB) therapy mainly aims at *PD-1*/*CTLA-4*, which can suppress the negative regulatory pathways and unleash T cells from the exhausted status. However, the expression levels of *PD-1* and *CTLA-4* failed to have significant differences between the no metastasis group and the lung metastasis group (**Supplementary Figure S2**), urging the need for further expanding treatment options. Drug repurposing of CMAP drugs for the lung metastasis was performed with the lung metastasis-related genes identified above and the CMAP perturbation signatures. The top 20 possible drugs that could reverse the breast cancer lung metastasis signature were presented (**Figure 6**), including *IGF-1* inhibitor, *mTOR* inhibitor, *PI3K* inhibitor, *SRC* inhibitor, aurora kinase inhibitor, and *JAK* inhibitor. In addition, FDA-approved drugs targeted for lung metastasis-related IRGs were listed (**Table 2**) (34).

**Table 2:** FDA-approved drugs targeted for lung metastasis-related IRGs.

Gene target	Effect	Drug	Reference
AKT1	Inhibition	Vemurafenib	CIViC
		GSK2141795	Hescheler et al. (34)
		AZD5363	
AR	Activation	DHT	Hickey et al. (40)
		Enobosarm	
EGFR	Inhibition	Gefitinib, Erlotinib	Hescheler et al. (34)
MET	Inhibition	Onartuzumab Crizotinib Foretinib Capmatinib Crizotinib, Vemurafenib Cabozantinib Crizotinib, Cetuximab Cabozantinib, Capmatinib	Hescheler et al. (34)
PTGS2	Inhibition	Aspirin	

IRG, immune-related genes.

## Discussion

With the great advances in breast cancer treatments, the prognosis of early breast cancer patients has been ameliorated significantly. However, approximately one-fifth of breast cancer patients will suffer from distant metastasis, which is the major reason for breast cancer-induced death (6). It has been reported that the median survival time for breast cancer patients with lung metastases was 21 months (41). The disease is nearly incurable and irreversible once metastasis occurs. A better understanding of the molecular and cellular mechanisms underlying lung metastasis could improve the overall survival of these patients. Identifying effective predictive biomarkers to predict and alert the lung metastasis may be a profound breakthrough applied in clinical practice. Our study is the first one that aims to clarify the immune composition, hub genes, and mutational characteristics that drive breast cancer lung metastasis from published multi-omics databases, incorporating primary tumor data of 1067 breast cancer patients in four datasets. The immune composition and tumor microenvironment of tumors that would metastasis to the lung were distinct from those that would not metastasis. In addition, tumors that metastasized to the lung have more aggressive malignant behaviors, higher mutation loads, and higher immunogenicity, which may be affected by the regulation of immune cells. We identified mast cell fraction as a prediction index of the status of lung metastasis in breast cancer patients. The low mast cell fraction defined a subtype of breast tumors that were highly proliferative, with higher mutation burdens, and were prone to have lung metastasis. We bring forward the idea that breast cancer patients with lung metastasis have distinct molecular and cellular characteristics and mutation profiles, which could differentiate them from those without lung metastasis.

There are views indicating that tumor metastasis is the outcome of the accumulation of mutations, especially mutations in metastasis-related genes (14). Higher mutation burdens correspond to worse survival in metastatic breast cancer patients (14). Tumor mutation burden is largely attributed to genomic instability. Genomic instability generates tumor heterogeneity, from which aggressive variants with strong metastatic ability can form secondary lesions (42). However, the association between mutations and neoantigen burdens of primary breast tumors and the outcomes of lung metastasis remains unknown. We found that there was no difference in the mutation load between the breast cancer patients with or without lung metastasis. SCNA and CTA levels were higher in the lung metastasis group, while the diversity of TCR was decreased compared to the no metastasis group. Generally speaking, breast cancer patients that developed lung metastasis later tended to have more malignant characteristics yet failed to activate more intense immune responses. However, many parameters failed to reach significant levels between the two groups, possibly due to the uneven numbers of the two groups.

*TP53* was the most frequently mutated gene in the lung metastasis group and it was significantly mutated more frequently in the lung metastasis group than in the no metastasis group. The expression level of *TP53* was higher in the no metastasis group (**Supplementary Table 4**). Therefore, the tumor suppressor gene *TP53* has a higher mutation rate and a lower expression level in the lung metastasis group. *MDN1* is also a tumor suppresser gene (43), which had higher mutation rate and elevated expression in the no metastasis group in the METABRIC cohort. *FLG2*, *FMN2*, and *ERBB3* have been reported to be related to breast cancer (44–46). We believed that further functional experiments are needed to prove the exact roles of these mutations. We tried to use TP53 as an example to prove that our analysis method was right, and we also listed other differentially mutated genes, hoping to inspire future studies.

The accumulation of mast cells has been reported to be related to poor prognosis in gastric, colorectal, and pancreatic tumors, while it remains controversial in breast cancer, demonstrating both pro-and anti-tumor roles (16). In our study, we found mast cell fraction can be used as a predictive index. The low mast cell content indicated a subtype of tumors that were more malignant, had higher immunogenicity and were prone to develop lung metastasis. A study conducted in a cohort containing 4444 invasive breast cancer patients with long-term follow-up showed that mast cell infiltration in invasive breast cancer could be served as an independent good prognostic marker, independent of tumor grade, age, tumor size, ER and Her2 status, and lymph node (47). Mast cells have been reported to stimulate estrogen receptor activity in breast cancer cells, which promoted the luminal phenotype, the less aggressive cancer types, and possibly explained the association between a higher mast cell infiltration and a better disease prognosis (48, 49). *In vivo* model using mast cell-depleting agent imatinib mesylate has shown accelerated tumor growth in a murine model of breast carcinoma, indicating an anti-tumor role of mast cells (50). However, mast cell can facilitate tumor angiogenesis and lymphangiogenesis and is positively correlated with lymph node metastasis (16). *In vivo* mast cell-depleting models have shown controversial results. Compared to their littermate controls, *c-Kit*-deficient mice showed significantly lighter tumor burden and refrained metastatic potential (15). Therefore, there is no clear verdict on this ongoing debate yet. These conflicting data suggested that mast cells might play different roles at different stages of breast cancer progression. Studies of mice models suggested that mast cells facilitated the growth and metastasis of tumors at the early stage, yet elevated mast cell infiltration in advanced breast tumors in humans could suppress breast cancer development and improve prognosis (15).

Drugs that inhibit mast cells activation and degranulation have been reported extensively in allergy and inflammatory diseases, while drugs that can activate mast cells remain a large void. Sorafenib has been reported to significantly increase the number and degranulation of skin-type mast cells in a stem cell factor (SCF) dependent manner (51). The effects of sorafenib on breast cancer mainly focus on breast cancer cells. Sorafenib inhibited the cell viability, migration, and invasion of breast cancer cells *in vitro* (52). Though a series of Phase IIb screening trials suggested the PFS benefit for sorafenib plus capecitabine as first- or second-line treatment for patients with HER2-negative advanced breast cancer, the Phase III RESILIENCE trial showed the combination of sorafenib with capecitabine did not improve PFS, OS, or the overall response rate in patients with HER2-negative advanced breast cancer and the rates of Grade 3 toxicities were higher in the sorafenib arm (53, 54). The drug delivery pattern and the subject selection may influence the outcome. An injectable double-layer-gel matrix of sorafenib and anti-CD47 antibody efficiently prevented tumor recurrence and metastasis in *in vivo* 4T1 mice models (55). The modified delivery pattern can reduce drug dose and side effects than oral intake. The participants can be chosen as patients with low mast cell infiltrations and high possibilities of lung metastasis as this study suggested. We hoped these novel molecular and genomic-driven therapeutic strategies can benefit the prognosis of breast cancer patients with lung metastasis.

A recent study conducted by Davis et al. reported that pharmacological blockade of oxidative phosphorylation significantly decreased metastatic load in the lungs in two breast cancer metastasis models, which highlighted its potential as a therapeutic target to prevent lung metastatic spread in breast cancer patients (5). We can recommend the patient who has a high probability to develop lung metastasis in the future to take a thorough inspection of the lung and apply advanced treatments that may appear in the near future. Therefore, risk evaluation plays a crucial role in making effective therapeutic strategies and follow-up management in breast cancer patients. The meta-analysis of 1067 patients showed that those with lower mast cell fractions exhibited higher risks of lung metastasis than those with higher mast cell fractions. The AUCs of mast cell fractions for lung metastasis prediction were comparable or even superior to other parameters used in metastasis prediction like tumor size, positive lymph nodes number, grade, TNM staging system, and signature reported by another study (18). In clinical settings, mast cell infiltration could be counted by tryptase IHC staining of dissected tumor samples or estimated by the xCell algorithm. The cut-off point was set to 25 mast cells per 10x field, which could be used to classify patients into two groups: low or high probability of lung metastasis. As for the xCell algorithm, the cut-off points in each dataset were not exactly the same, indicating that the specific cut-off value in future use needed to be confirmed by each institution. The reason may be that the different platforms, demographic heterogeneity, and doctors’ preferences would all affect the choice of cut-off point, just like many reference values for clinical and laboratory indexes are different in each hospital. We stated that adopting mast cell fraction could ameliorate the prediction accuracy and thereby improving the prognosis of breast cancer patients.

Our research had several limitations. Firstly, the limited number of lung metastasis compared to no metastasis in the cohorts might influence the statistic power. The number of mast cells is related to the molecular subtypes of breast cancer, but the roles of molecular subtypes have not been discussed in detail in this study due to the limited number of events and the lack of molecular subtype information in one dataset. Secondly, in the METABRIC dataset, although the predictive power of mast cell fraction is comparable to the existing parameters, yet it is still relatively weak. Last but not least, experiments for revealing the underlying mechanism of mast cells in attenuating lung metastasis are needed in subsequent studies.

In the present study, we used four public datasets with matched clinical and mRNA data to reveal the distinct microenvironment and immune composition between patients with or without lung metastasis. We used multi-omics data of the TCGA cohort to emphasize the following characteristics that could contribute to lung metastasis: more aggressive tumor malignant behaviors, severer genomic instability, higher immunogenicity but showed generalized inhibition of effector functions of immune cells. Furthermore, we found mast cell fraction can be used as a parameter for individual lung metastasis status prediction. When the mast cell content was low, the tumor was more aggressive and prone to have lung metastasis. This finding may apply to all patients because it is based on a large-scale cohort. As far as we know, this study is the first comprehensive analysis of the molecular and cellular characteristics and mutation profiles of breast cancer metastasis to the lung. We believe these findings might be suitable for prognostic prediction for breast cancer patients and provide novel genomic-driven therapeutic strategies for breast cancer metastasis to the lung.

## Conclusion

In our current study, we revealed that the microenvironment and immune composition between patients with or without lung metastasis were distinct. Breast tumors that developed lung metastasis had more aggressive malignant behaviors, severer genomic instability, higher immunogenicity but showed generalized inhibition of effector functions of immune cells. Among the immune cells, mast cell fraction can be used as an index for individual lung metastasis status prediction. When the mast cell content was low, the tumor was more aggressive and prone to have lung metastasis.

## Data Availability Statement

The datasets presented in this study can be found in online repositories. The names of the repository/repositories and accession number(s) can be found in the article/**Supplementary Material**.

## Ethics Statement

The studies involving human participants were reviewed and approved by the ethics review committee of the Second Affiliated Hospital of Zhejiang University School of Medicine. The patients/participants provided their written informed consent to participate in this study.

## Author Contributions

LZ conceived the presented idea. LZ and JP developed the methodology, acquired and analyzed the omics data. ZW, CY, WC, and JXJ collected human samples and performed staining analysis. ZZ, FJ, YZ, JHJ, KS, and GR searched and organized the literature. LZ wrote the manuscript. JP and JH edited the manuscript. All authors contributed to the article and approved the submitted version.

## Funding

This study was supported by the National Natural Science Foundation of China under Grant (No.81520108024, 81872317, 81930079) and Health Commission of Zhejiang Province under Grant (WKJ-ZJ-1803).

## Conflict of Interest

The authors declare that the research was conducted in the absence of any commercial or financial relationships that could be construed as a potential conflict of interest.

## Publisher’s Note

All claims expressed in this article are solely those of the authors and do not necessarily represent those of their affiliated organizations, or those of the publisher, the editors and the reviewers. Any product that may be evaluated in this article, or claim that may be made by its manufacturer, is not guaranteed or endorsed by the publisher.

## Supplementary Material

Supplementary Figure 1 Correlation matrix of the 51 types of cells.

Supplementary Figure 2 Comparison of the mRNA expression fold changes of immune-related genes and chemokines.

Supplementary Figure 3 Immunogenicity of breast cancer patients with or without lung metastasis in the TCGA cohort.

Supplementary Figure 4 Neoantigens of breast cancer patients with lung metastasis.

Supplementary Figure 5 GSEA analysis of breast cancer patients in the GSE2603 cohort.

Supplementary Figure 6 Mast cell fraction and survival.

Supplementary Figure 7 Correlation between pro-metastasis IRGs and mast cell fractions.

## Abbreviations

IRGs, immune-related genes; TNM, tumor node metastasis; METABRIC, Molecular Taxonomy of Breast Cancer International Consortium; TILs, tumor-infiltrating lymphocytes; TCGA, The Cancer Genome Atlas; GDC Data Portal, Genomic Data Commons Data Portal; GSEA, gene set enrichment analysis; WGCNA, weighted correlation network analysis; DEmRNAs, differentially expressed mRNAs; GEO, gene expression omnibus; SCNAs, somatic copy number alterations; HRD, homologous recombination defects; CTA, cancer testis antigens; IPS, immunophenoscore; TCR, T cell antigen receptor; TCIA, The Cancer Immunome Database; ROC, receiver operating characteristic curve; KM, Kaplan-Meier; AUC, area under the receiver operating characteristics curve; CMAP, connectivity map; GO, gene ontology; CTL, cytotoxic T lymphocyte; IFN, interferon; HLA, human leukocyte antigen; SNP, single nucleotide polymorphisms; SNV, single-nucleotide variant; ICB, immune checkpoint blockade; SEER, Surveillance, Epidemiology, and End Results; DC, dendritic cells; MPP, multipotent progenitors; T_em_, effector memory T cells; CMP, common myeloid progenitors; MEP, megakaryocyte erythroid progenitors; GMP, granulocyte-macrophage progenitors; Tregs, regulatory T cells; HSC, hematopoietic stem cells; T_cm_, central memory T cells; ly endothelial cells, lymphatic endothelial cells; mv endothelial cells, microvascular endothelial cells; MSC, mesenchymal stem cells; aDC, activated dendritic cells; pDC, plasmacytoid dendritic cells; cDC, conventional dendritic cells; iDC, immature dendritic cells; Th2 cells, type 2 T helper cells; CLP, common lymphoid progenitors; Th1 cells, type 1 T helper cells; NKT, natural killer T cells; Tgd cells, gamma delta T-cells; SCF, stem cell factor; PFS, progression free survival.
