# Supplementary figures and images for: Multi-Omics Profiling Suggesting Intratumoral Mast Cells as Predictive Index of Breast Cancer Lung Metastasis
Source: Front Oncol. 2022 Jan 17;11:788778. doi: 10.3389/fonc.2021.788778 (PMC8801492; doi:10.3389/fonc.2021.788778)

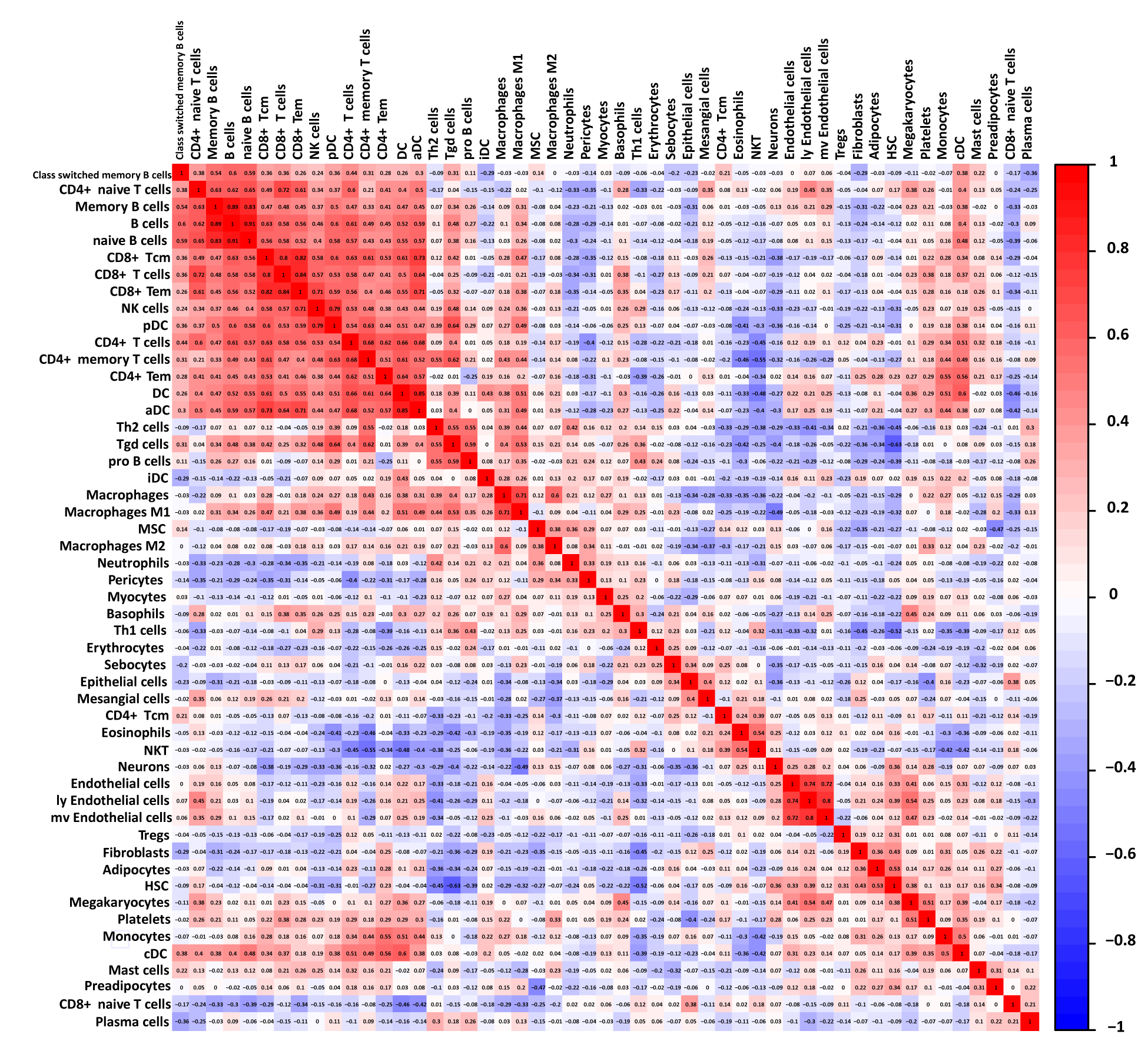

Supplement: Supplementary Figure 1 — Correlation matrix of the 51 types of cells. [file Image_1.tif]

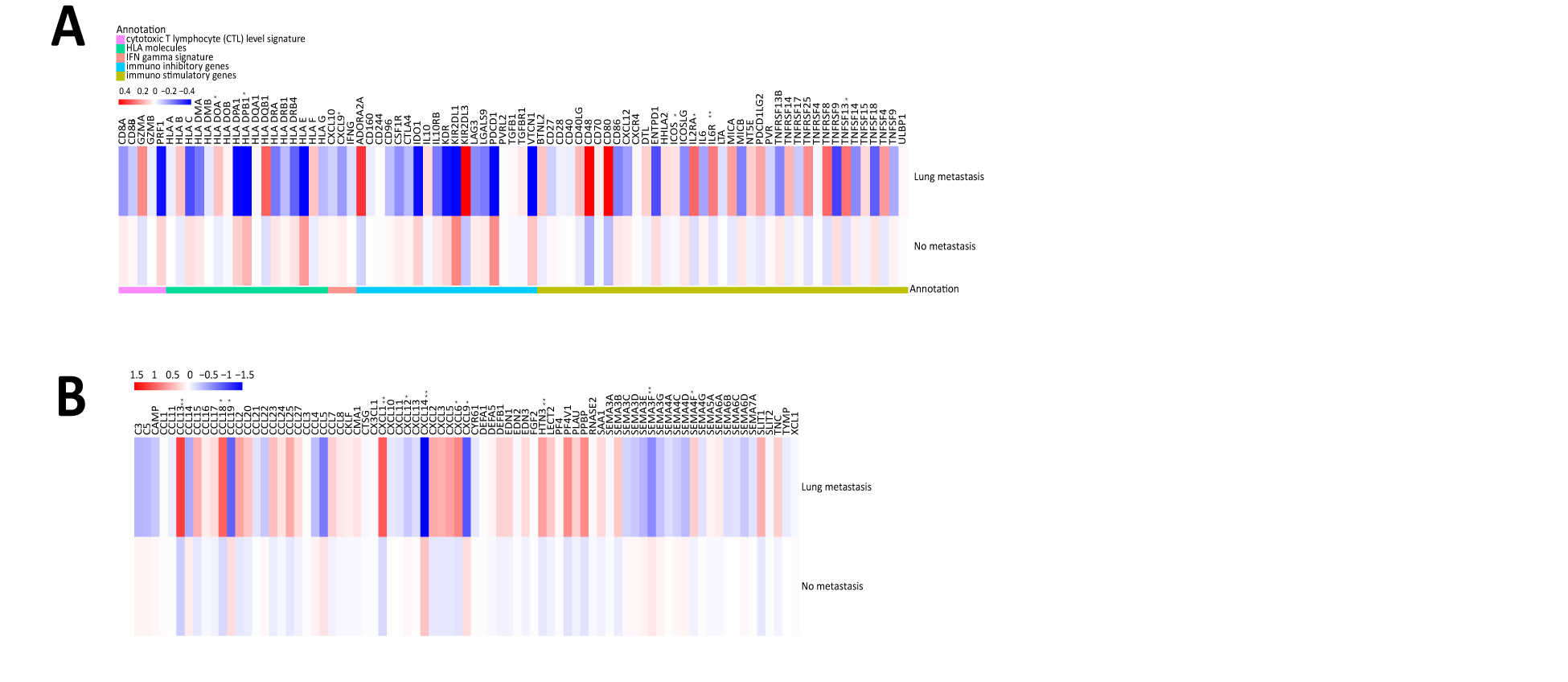

Supplement: Supplementary Figure 2 — Comparison of the mRNA expression fold changes of immune-related genes and chemokines. [file Image_2.tif]

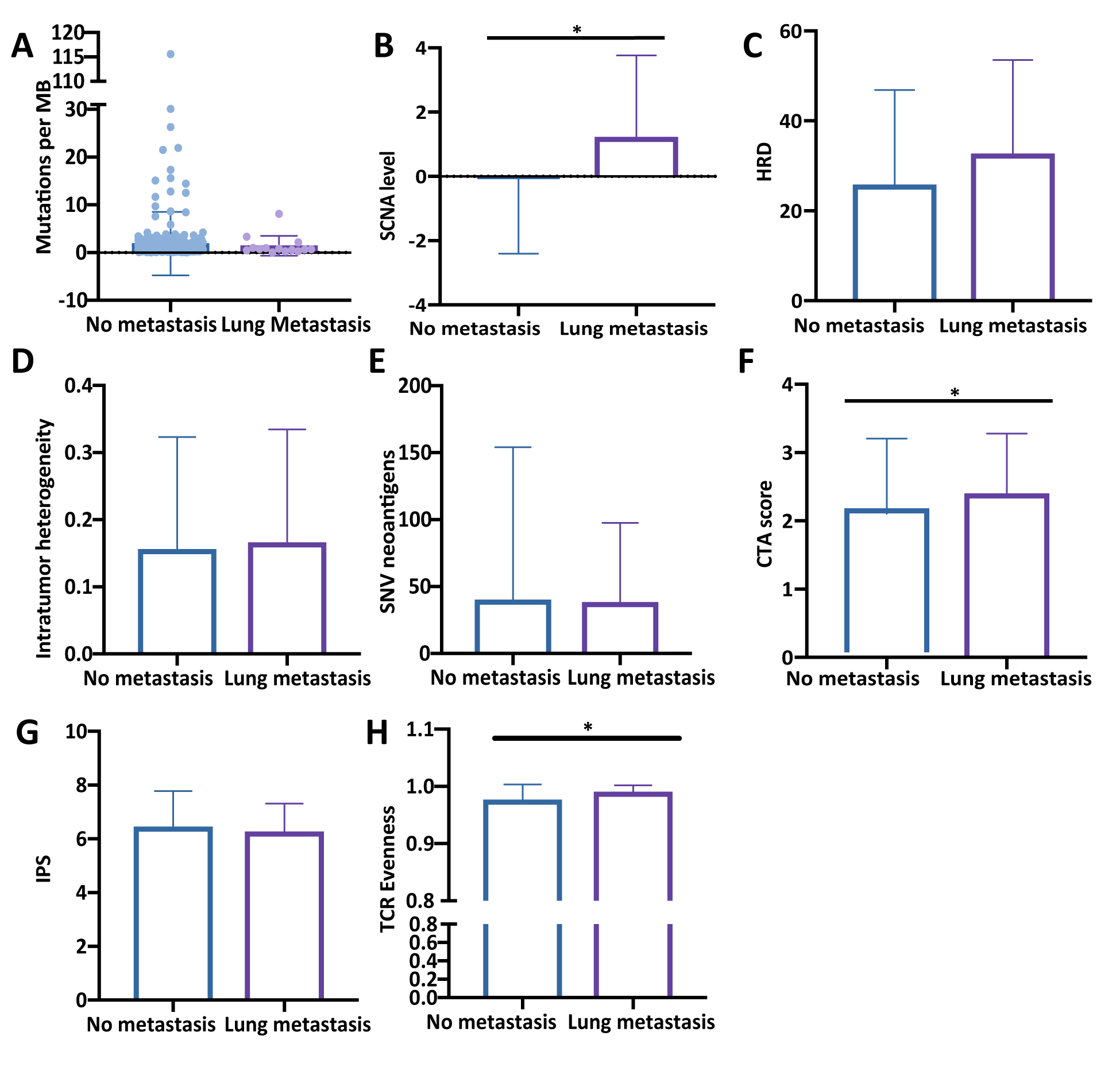

Supplement: Supplementary Figure 3 — Immunogenicity of breast cancer patients with or without lung metastasis in the TCGA cohort. [file Image_3.tif]

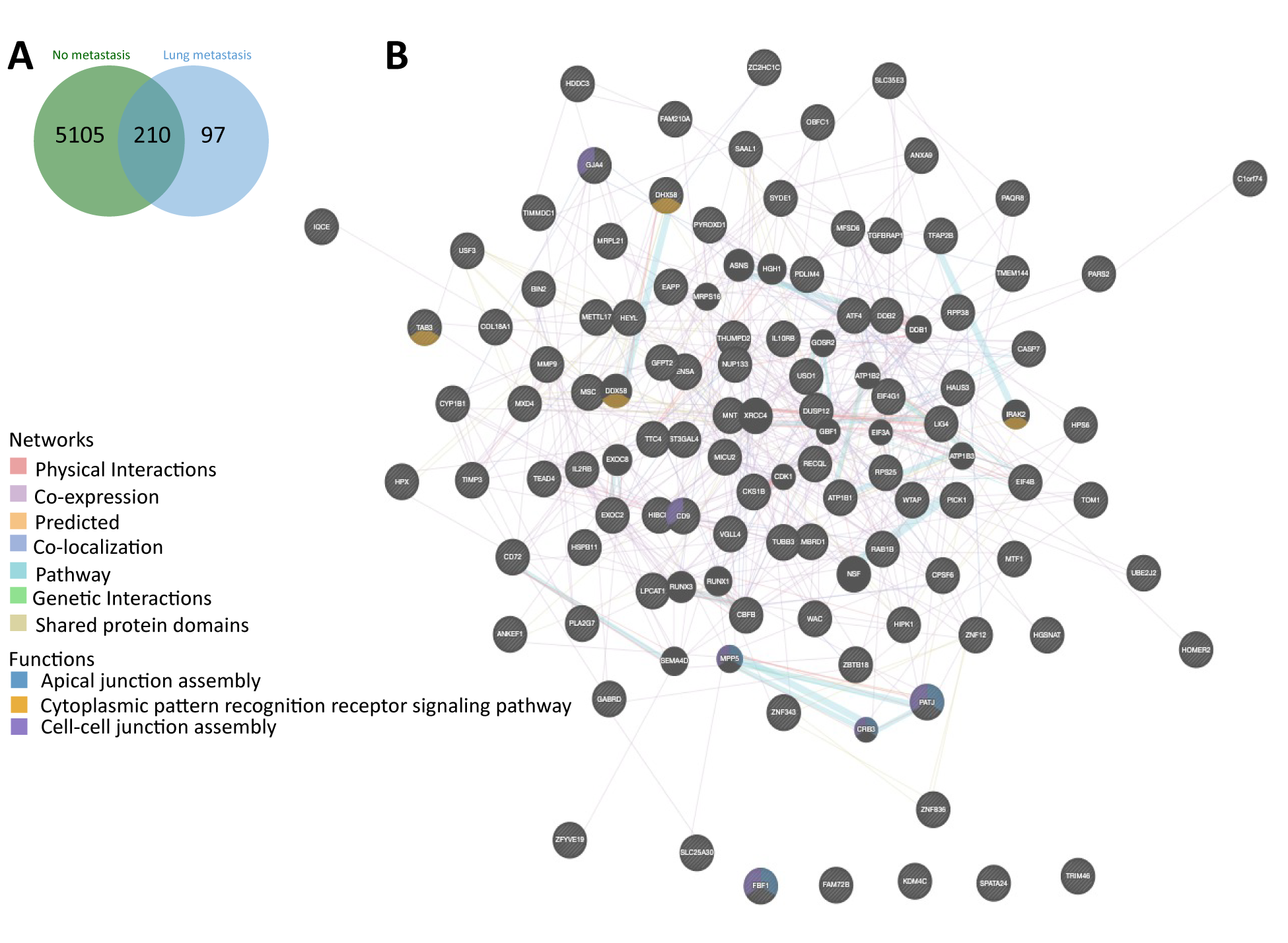

Supplement: Supplementary Figure 4 — Neoantigens of breast cancer patients with lung metastasis. [file Image_4.tif]

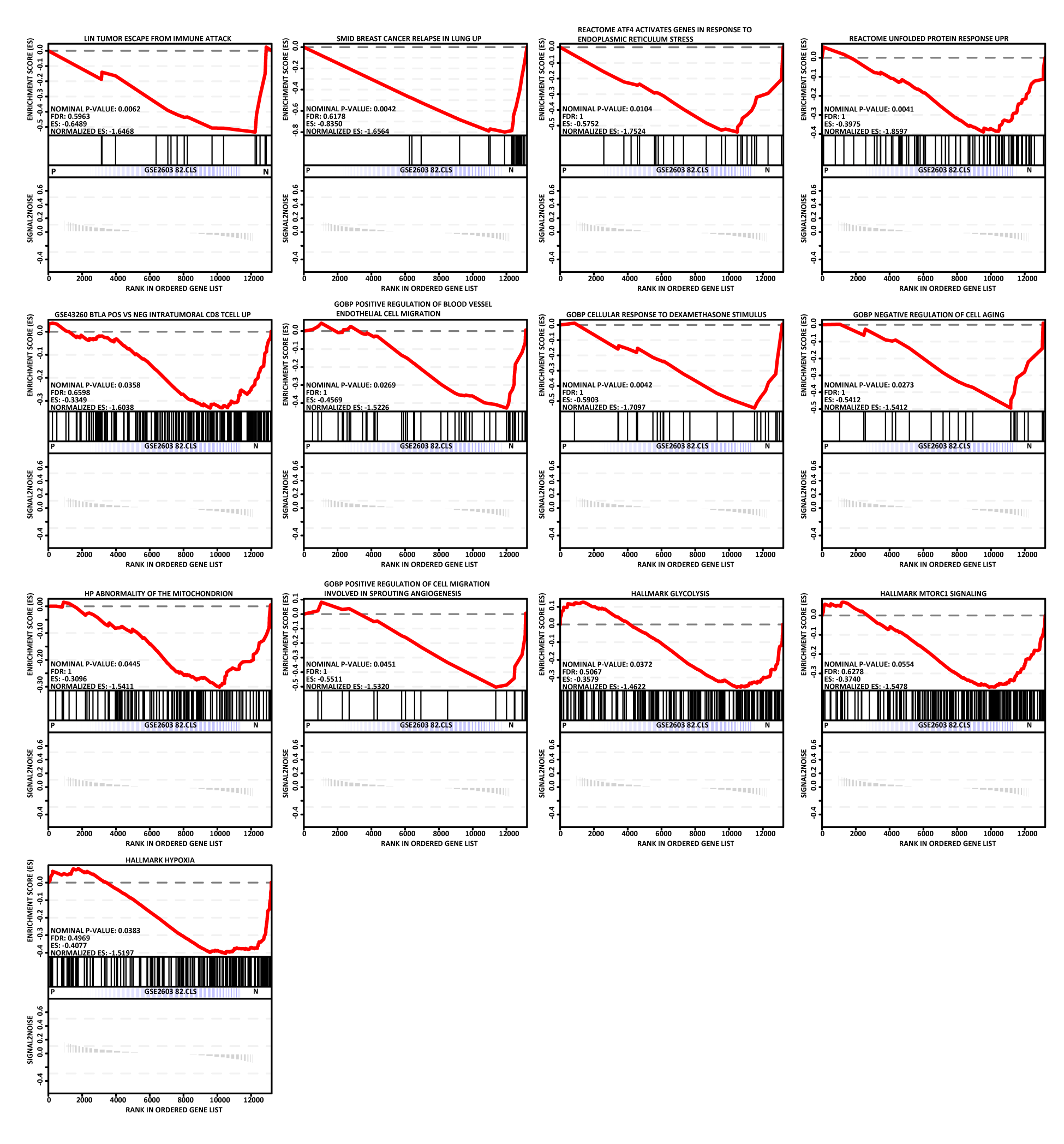

Supplement: Supplementary Figure 5 — GSEA analysis of breast cancer patients in the GSE2603 cohort. [file Image_5.tif]

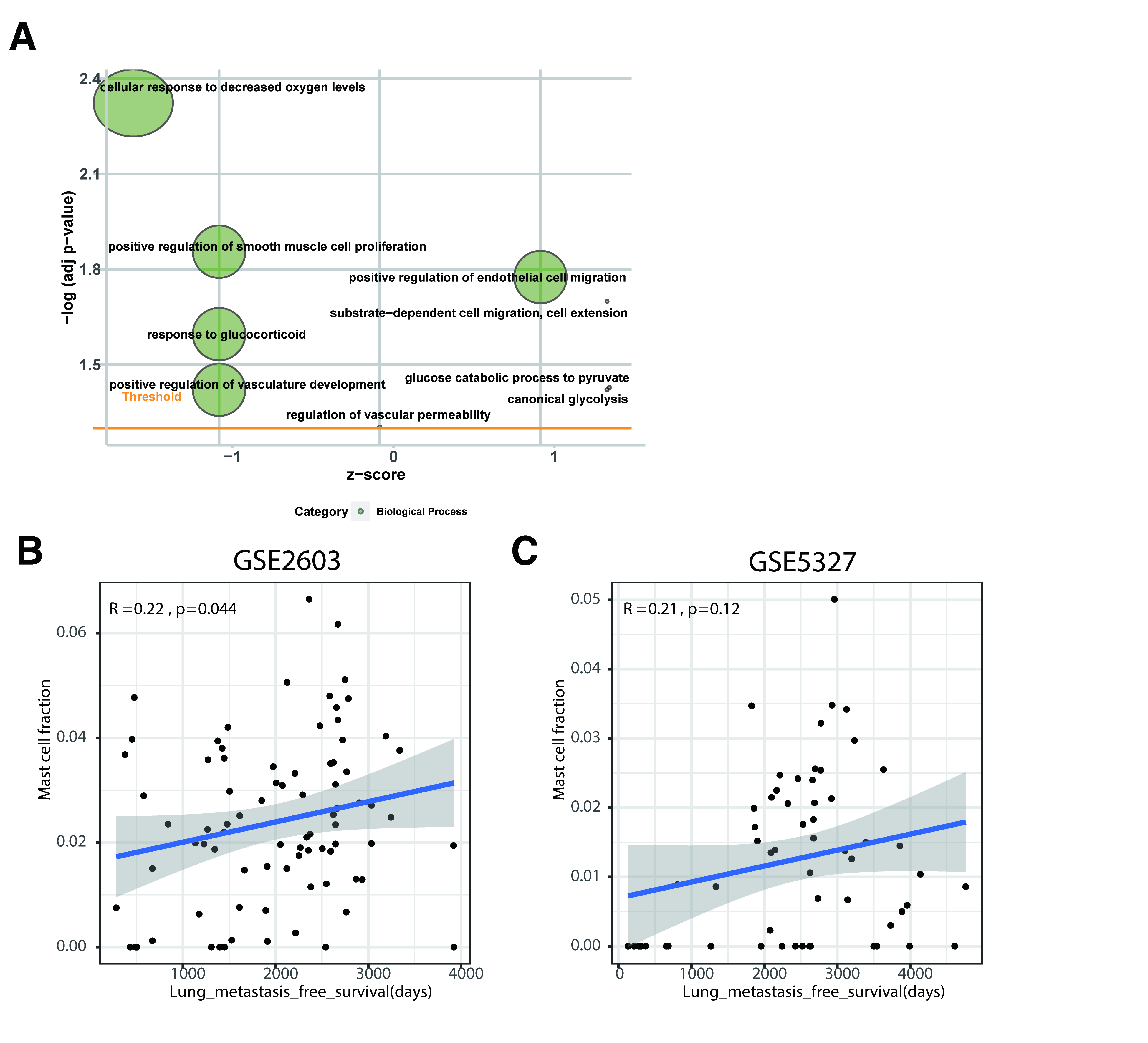

Supplement: Supplementary Figure 6 — Mast cell fraction and survival. [file Image_6.tif]

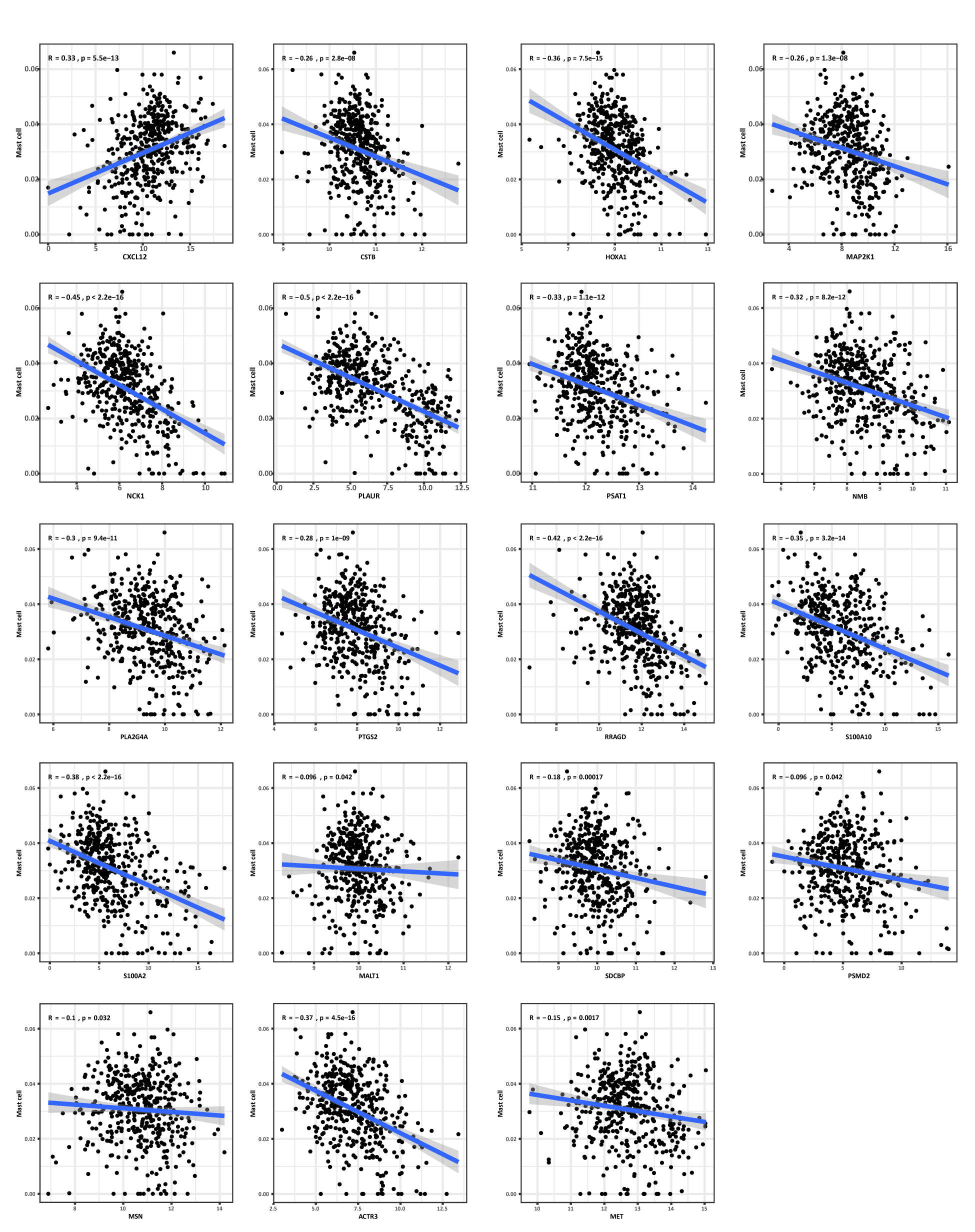

Supplement: Supplementary Figure 7 — Correlation between pro-metastasis IRGs and mast cell fractions. [file Image_7.tif]
